# Association of Neighborhood Deprivation with Stage at Diagnosis and Treatment Delay for Breast Cancer in Philadelphia

**DOI:** 10.1245/s10434-025-17367-w

**Published:** 2025-05-12

**Authors:** Isabel C. Yoon, Stephany Perez-Rojas, Bhavya Ancha, Leisha C. Elmore, Alina M. Mateo, Margaret S. Pichardo, Julia C. Tchou, Jennifer Q. Zhang, Rebecca A. Hubbard, Oluwadamilola M. Fayanju

**Affiliations:** 1 Perelman School of Medicine, University of Pennsylvania, Philadelphia, PA USA; 2 Division of Breast Surgery, Department of Surgery, Perelman School of Medicine, University of Pennsylvania, Philadelphia, PA USA; 3 Department of Medicine, The Johns Hopkins University, Baltimore, MD USA; 4 Rena Rowan Breast Center, Abramson Cancer Center, Penn Medicine, Philadelphia, PA USA; 5 Leonard Davis Institute of Health Economics (LDI), University of Pennsylvania, Philadelphia, PA USA; 6 Department of Biostatistics, Epidemiology and Informatics, Perelman School of Medicine, University of Pennsylvania, Philadelphia, PA USA; 7 Department of Biostatistics, Brown University School of Public Health, Providence, RI USA; 8 Penn Center for Cancer Care Innovation (PC3I), Abramson Cancer Center, Penn Medicine, Philadelphia, PA USA

## Abstract

**Introduction:**

Neighborhood deprivation and other sociodemographic factors are associated with breast cancer outcomes, but in Philadelphia, the country’s poorest large city, these factors have been understudied. We examined their association with stage at breast cancer diagnosis and treatment delay (>60 days after diagnosis).

**Methods:**

We identified women aged ≥18 years with breast cancer at an academic health system based in Philadelphia from 2011 to 2019. The Area Deprivation Index (ADI) was calculated across the cohort and grouped into quartiles: ADI 1 = least deprived, ADI 4 = most deprived. Multivariable logistic regression estimated sociodemographic associations with advanced stage (III–IV) at diagnosis and treatment delay.

**Results:**

Overall, 11,108 patients were identified. White patients constituted a larger proportion of the least versus most deprived group (ADI 1 = 84.4% vs. ADI 4 = 50.9%), while the proportion of Black patients was highest in the most deprived group (ADI 1 = 3.9% vs. ADI 4 = 41.5%). Patients in the ADI 4 group (vs. ADI 1; odds ratio [OR] 1.48, 95% confidence interval [CI] 1.19–1.84), who identified as Black (vs. White; OR 1.35, 95% CI 1.11–1.63), and with Medicaid insurance (OR 1.94, 95% CI 1.51–2.49) or no insurance (OR 2.21, 95% CI 1.27–3.67) versus privately insured patients had higher odds of presenting with advanced stage (all *p* < 0.05). Patients who identified as Asian, had Medicaid insurance or no insurance, were >70 years of age, and presented with advanced stage were less likely to receive treatment within 60 days, while patients in the ADI 2–4 group were twice as likely to receive treatment within 60 days as patients in the ADI 1 group.

**Conclusions:**

Neighborhood deprivation was associated with advanced stage at presentation, but not treatment delay, for patients with breast cancer in the Philadelphia metropolitan area, suggesting neighborhood-level opportunities to facilitate screening and more early-stage diagnoses.

Neighborhood deprivation, a metric describing a neighborhood’s relative number of resources, is a composite variable that captures multiple sociodemographic variables, such as income, employment, education, insurance status, and housing. A growing body of research has demonstrated an association between neighborhood deprivation and worse cancer outcomes, including worse survival, diagnosis at a more advanced stage, and early discontinuation of therapy.^1–4^

Neighborhood deprivation with regard to breast cancer outcomes has been increasingly studied across various states. Studies conducted in New Jersey, Ohio, and South Carolina each demonstrated an association between neighborhood and specific measures of breast cancer outcomes.^4–7^ For example, a study conducted in New Jersey described how increases in the physical disorder of a neighborhood were associated with higher odds of being diagnosed with late-stage breast cancer. Findings from such studies have also underscored the complex interplay of population-level variables, such as neighborhood deprivation, and individual-level characteristics. The study by Plascak and colleagues found an association between residing in non-redlined neighborhoods and more favorable breast cancer outcomes, a relationship that was only found for non-Latina White women.^5^

As the poorest large city in the United States (US), Philadelphia is an important setting for neighborhood deprivation studies and public health interventions. Data from the US Census’ American Community Survey in 2019 demonstrated that 23% of the population in Philadelphia lived below the federal poverty line, double the national average. Furthermore, Philadelphia remains highly segregated, with poverty being most concentrated in North and West Philadelphia.^8^ Inequities in health are also pervasive in Philadelphia. A 2019 report by the Department of Public Health and Drexel University found large variations in health outcomes between neighborhoods. Notably, neighborhood-level life expectancy for men ranged from a high of 82 years to a low of just under 64 years.^9^ Recent geographic studies have added further understanding, demonstrating differences in postoperative outcomes, cancer rates, chronic disease, and maternal outcomes based on location.^10–12^

There is a relative paucity of literature specifically assessing breast cancer outcomes in Philadelphia, where, in keeping with national trends, breast cancer is the most common malignancy among women and the second leading cause of female cancer mortality.^13^ Accordingly, we sought to examine the association between social determinants of health, including neighborhood deprivation, on stage of breast cancer at diagnosis and time to treatment (TTT).

## Methods

### Data Source

The Penn Medicine Cancer Registry is a database consisting of patient-level data from all patients with a cancer diagnosis who were evaluated within the University of Pennsylvania Health System (UPHS). The Breast Cancer Tumor Registry dataset, a subset of this Penn Medicine Cancer Registry, was used for this study. This study operated under a waiver of the Health Insurance Portability and Accountability Act (HIPAA) after being reviewed by the University of Pennsylvania Institutional Review Board. The study followed the Strengthening the Reporting of Observational Studies in Epidemiology (STROBE) Statement guidelines for reporting observational studies.^14^

### Study Population

In this retrospective cohort study conducted at a single academic health system, we identified women ≥18 years of age diagnosed with and treated for stage 0–IV breast cancer between 1 January 2011 and 31 December 2019, at one of the following UPHS facilities serving patients in the Philadelphia metropolitan area, which includes communities in Delaware, New Jersey, and Pennsylvania: Hospital of University of Pennsylvania (HUP), Pennsylvania Hospital (PAH), Penn Presbyterian Medical Center (PPMC), Chester County Hospital (CCH), Lancaster General Health (LGH), and Penn Medicine Princeton Health (PMPH). There were no exclusion criteria pertaining to area of residence or community type.

Patients were excluded from the entire analysis if they were missing any of the following variables: clinical stage, diagnosis facility, and treatment facility. Patients with stage 0 disease (i.e., ductal carcinoma in situ [DCIS]) are reported in Table 1 but were excluded from the analyses given differences in treatment trajectory and outcomes for non-invasive carcinoma as compared with invasive disease.

### Predictors

#### Area Deprivation Index (ADI)

Using zip codes derived from the cancer registry and the 2021 Neighborhood Atlas, we determined the ADI for all patients included in our study. The ADI is a measure that assigns each census block group a national ranking percentile from 1 to 100, with 1 indicating neighborhoods within block groups of lowest levels of disadvantage and 100 indicating highest levels of disadvantage.^15^

We first performed data preprocessing on the Area Deprivation Index (ADI) variable. The ADI variable was originally a right-skewed continuous variable with levels ranging from 1 to 100. The overall median ADI score was 32 (average 37, interquartile range [IQR] 16–50). There was a greater representation of patients living in neighborhoods of ADI <50 than those living in neighborhoods with ADI >50 (8260 vs. 2848). To normalize the variable, ADI scores were grouped into four levels using data binning: lowest ADI scores (least deprived, quartile 1) to highest ADI scores (most deprived, quartile 4). Due to the spread of the data, quartiles were deemed the most appropriate subgroup level. The differences between ADI groups were minimized, and each cohort had an approximately even number of patients.

#### Covariates

Data extracted from the Breast Cancer Tumor Registry included age, race and ethnicity, marital status, year of diagnosis, clinical stage at diagnosis, body mass index (BMI), type of insurance, and type of treatment received. Race was self-reported by patients upon new patient intake, selecting from White, Black, Asian, Native Hawaiian or Pacific Islander, or other/unknown. Ethnicity was also self-reported by patients as Hispanic or non-Hispanic.

For data analysis, race and ethnicity were categorized into the following per the 2021 *Journal of the American Medical Association* (JAMA) guidelines on reporting race and ethnicity: non-Hispanic White (hereby referred to as ‘White’), non-Hispanic Black (‘Black’), Asian and Pacific Islanders (‘Asian’), and Hispanic.^16^ Insurance type extracted from the registry was categorized as private, Medicaid, Medicare, military, uninsured, and unknown.

### Outcomes

The primary outcomes assessed were stage at time of diagnosis and TTT. Stage at diagnosis was defined as clinical stage I–IV based on the 7th edition of American Joint Committee on Cancer (AJCC) staging system. Advanced stage at diagnosis was defined as clinical stages III and IV.

The TTT variable was defined as the number of days between the date of diagnosis to the date of receiving treatment. Treatment was defined to include surgery, radiation, chemotherapy, immunotherapy, or endocrine therapy.

### Statistical Analysis

Descriptive analyses were summarized as number (%) in Table 1 and stratified by ADI. The *p*-values were derived using a Chi-square test. Multivariable logistic regression models, calculated via generalized linear models, estimated associations of ADI and patient-level variables such as age, race and ethnicity, and type of insurance with stage at diagnosis.

Analyses of variance were conducted separately on TTT since diagnosis, and stage at diagnosis, to see whether there were differences within patient-level variables, including stage at diagnosis, ADI rank, race and ethnicity groups, age at diagnosis, and insurance type.

Two different multivariable logistic regression linear models were used to estimate the associations. The first model estimated the association of stage at diagnosis (low [I–II] vs. high [III–IV]) and patient-level variables such as age, race/ethnicity, and insurance type. The second model estimated the association of treatment within 60 days and patient-level variables such as age, race/ethnicity, insurance type, and stage at diagnosis. Odds ratios (ORs) and their corresponding *p*-values are presented in Tables 1, 2, 3 and 4.

We aimed to reduce selection bias due to possible associations between stage and treatment, as well as a possible association with stage and ADI, by only including patients who were both diagnosed and treated at our health system. This better ensured for a random sample of breast cancer diagnosis regardless of stage.

A sensitivity analysis was conducted for the removal of patients with a missing zip code, drawing the line at 5% change (95% confidence interval [CI]). This was also done for collapsing variables. After carrying out these sensitivity analyses, the results did not meaningfully change.

## Results

### Study Population

**Fig. 1:**
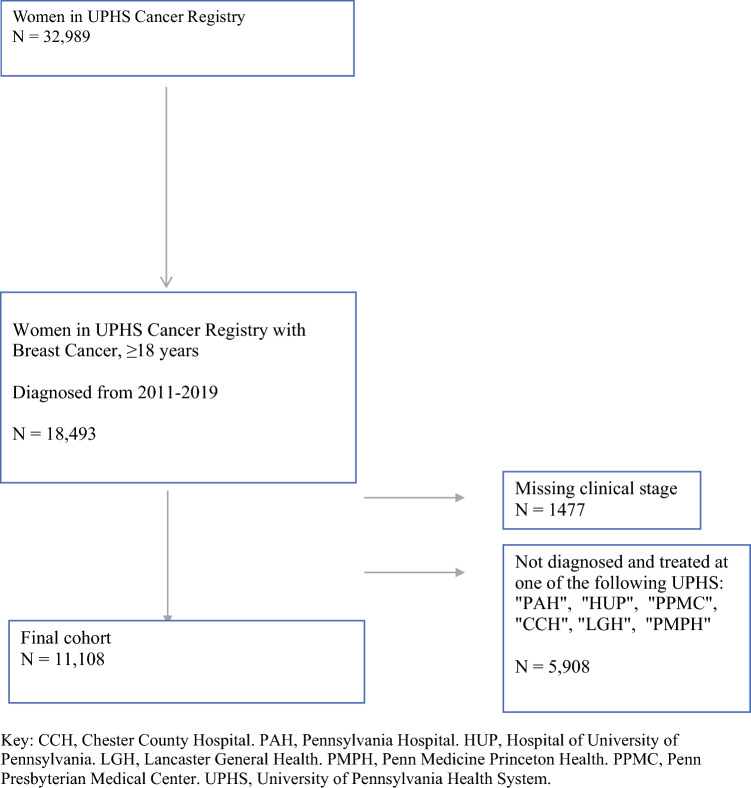
Cohort selection. *CCH* Chester County Hospital, *PAH* Pennsylvania Hospital, *HUP* Hospital of University of Pennsylvania, *LGH* Lancaster General Health, *PMPH* Penn Medicine Princeton Health, *PPMC* Penn Presbyterian Medical Center, *UPHS* University of Pennsylvania Health System

Overall, 11,108 women (White, *n* = 8529 [76.8%]; Black, *n* = 1695 [15.3%]; Asian, 455 [4.1%]; Hispanic, *n* = 298 [2.7%]; and other 131 [1.2%]) were identified in the Penn Tumor Registry and met the inclusion criteria (Fig. 1). There were 2743 women in the ADI 1 group, 2582 in the ADI 2 group, 2852 in the ADI 3 group, and 2931 in the ADI 4 group.

All ADI groups had similar age distributions, with the majority of women in each group being 40–70 years of age. The proportion of White women decreased from ADI 1 (ADI 1 = 84.4%) to ADI 4 (ADI 4 = 50.9%), which was also seen for Asian women (ADI 1 = 8.5%, ADI 4 = 1.5%). Conversely, the proportion of Black (ADI 1 = 3.9%, ADI 4 = 41.5%) and Hispanic (ADI 1 = 1.5%, ADI 4 = 5.0%) women increased with increased neighborhood deprivation (Table 1).

**Table 1:** Women diagnosed with breast cancer at Penn Medicine, 2011–2019, stratified by ADI

	ADI 1 [*n *= 2743]	ADI 2 [*n *= 2582]	ADI 3 [*n *= 2852]	ADI 4 [*n *= 2931]	Total [*N* = 11,108]	*p*-Value
Age, years						<0.001
<40	113 (4.1)	129 (5.0)	120 (4.2)	169 (5.8)	531 (4.8)	
40–70	2014 (73.4)	1859 (72.0)	1942 (68.1)	1941 (66.2)	7756 (69.8)	
>70	616 (22.5)	594 (23.0)	790 (27.7)	821 (28.0)	2821 (25.4)	
Race and ethnicity						<0.001
Asian	234 (8.5)	105 (4.1)	71 (2.5)	45 (1.5)	455 (4.1)	
Hispanic	41 (1.5)	43 (1.7)	67 (2.3)	147 (5.0)	298 (2.7)	
Black	106 (3.9)	179 (6.9)	193 (6.8)	1217 (41.5)	1695 (15.3)	
White	2316 (84.4)	2223 (86.1)	2497 (87.6)	1493 (50.9)	8529 (76.8)	
Other	46 (1.7)	32 (1.2)	24 (0.8)	29 (1.0)	131 (1.2)	
Marital status						<0.001
Divorced	23 (0.8)	36 (1.4)	130 (4.6)	98 (3.3)	287 (2.6)	
Married	175 (6.4)	203 (7.9)	197 (6.9)	350 (11.9)	925 (8.3)	
Separated	1976 (72.0)	1630 (63.1)	1632 (57.2)	1102 (37.6)	6340 (57.1)	
Single (never married)	266 (9.7)	338 (13.1)	351 (12.3)	738 (25.2)	1693 (15.2)	
Unmarried or domestic partner	7 (0.3)	10 (0.4)	7 (0.2)	6 (0.2)	30 (0.3)	
Widowed	230 (8.4)	262 (10.1)	297 (10.4)	436 (14.9)	1225 (11.0)	
Unknown	54 (2.0)	84 (3.3)	220 (7.7)	144 (4.9)	502 (4.5)	
Insurance						<0.001
Private	2152 (78.5)	1858 (72.0)	1677 (58.8)	1702 (58.1)	7389 (66.5)	
Medicare	435 (15.9)	476 (18.4)	637 (22.3)	551 (18.8)	2099 (18.9)	
Medicaid	26 (0.9)	58 (2.2)	110 (3.9)	390 (13.3)	584 (5.3)	
No insurance	37 (1.3)	32 (1.2)	32 (1.1)	23 (0.8)	124 (1.1)	
Military	9 (0.3)	9 (0.3)	8 (0.3)	15 (0.5)	41 (0.4)	
Missing	84 (3.1)	149 (5.8)	388 (13.6)	250 (8.5)	871 (7.8)	
BMI, kg/m^2^						<0.001
Underweight (<18.5)	32 (1.2)	16 (0.6)	10 (0.4)	18 (0.6)	76 (0.75)	
Normal weight (18.5–24.9)	578 (21.1)	509 (19.7)	253 (8.9)	256 (8.7)	1596 (14.4)	
Overweight (25.0–29.9)	379 (13.8)	399 (15.5)	243 (8.5)	368 (12.65)	1389 (12.5)	
Obese (30.0–35.0)	180 (6.6)	240 (9.3)	159 (5.6)	391 (13.3)	970 (8.7)	
Morbidly obese (>35.0)	111 (4.0)	174 (6.7)	114 (4.0)	365 (12.5)	764 (6.9)	
Unknown	1463 (53.3)	1244 (48.2)	2073 (72.7)	1533 (52.3)	6316 (56.8)	
Diagnosis year						0.003
2011	302 (11.0)	251 (9.7)	299 (10.5)	276 (9.4)	1128 (10.2)	
2012	274 (10.0)	247 (9.6)	280 (9.8)	320 (10.9)	1121 (10.1)	
2013	298 (10.9)	271 (10.5)	279 (9.8)	307 (10.5)	1155 (10.4)	
2014	324 (11.8)	278 (10.8)	315 (11.0)	340 (11.6)	1257 (11.3)	
2015	370 (13.5)	295 (11.4)	320 (11.2)	313 (10.7)	1298 (11.7)	
2016	316 (11.5)	295 (11.4)	318 (11.2)	336 (11.5)	1267 (11.4)	
2017	338 (12.3)	320 (12.4)	360 (12.6)	373 (12.7)	1391 (12.5)	
2018	248 (9.0)	297 (11.5)	349 (12.2)	341 (11.6)	1235 (11.1)	
2019	273 (10.0)	326 (12.6)	332 (11.6)	325 (11.1)	1256 (11.3)	
Clinical stage at diagnosis						<0.001
0	711 (25.9)	603 (23.4)	615 (21.6)	655 (22.3)	2584 (23.3)	
I	1374 (50.1)	1252 (48.5)	1391 (48.8)	1318 (45.0)	5335 (48.0)	
II	486 (17.7)	514 (19.9)	594 (20.8)	623 (21.3)	2217 (20.0)	
III	75 (2.7)	103 (4.0)	140 (4.9)	165 (5.6)	483 (4.3)	
IV	75 (2.7)	110 (4.3)	112 (3.9)	170 (5.8)	489 (4.4)	
Average days to time to treatment [SD]	46.09 [25.26]	49.56 [25.26]	49.74 [31.66]	49.29 [37.74]		0.013
Surgery						0.952
Yes	2548 (92.9)	2412 (93.4)	2693 (94.4)	2715 (92.6)	10,368 (93.3)	
No	195 (7.1)	170 (6.6)	159 (5.6)	216 (7.4)	740 (6.7)	
Radiation therapy						0.652
Yes	1476 (53.8)	1429 (55.3)	1623 (56.9)	1582 (54.0)	6110 (55.0)	
No	1267 (46.2)	1153 (44.7)	1229 (43.1)	1349 (46.0)	4998 (45.0)	
Immunotherapy						0.042
Yes	187 (6.8)	204 (7.9)	247 (8.7)	238 (8.1)	876 (7.9)	
No	2556 (93.2)	2378 (92.1)	2605 (91.3)	2693 (91.9)	10,232 (92.1)	
Chemotherapy						0.005
Yes	748 (27.3)	770 (29.8)	832 (29.2)	911 (31.1)	3261 (29.4)	
No	1995 (72.7)	1812 (70.2)	2020 (70.8)	2020 (68.9)	7847 (70.6)	
Endocrine therapy						0.240
Yes	1708 (62.3)	1686 (65.3)	1848 (64.8)	1758 (60.9)	7027 (63.3)	
No	1035 (37.7)	896 (34.7)	1004 (35.2)	1146 (39.1)	4081 (36.7)	
Type of treatment received first						0.045
Surgery first	2384 (86.9)	2224 (86.1)	2524 (88.5)	2543 (86.8)	9675 (87.1)	
Chemotherapy/immunotherapy first	167 (6.1)	184 (7.1)	173 (6.1)	175 (6.0)	699 (6.3)	
Endocrine therapy first	68 (2.5)	96 (3.7)	83 (2.9)	114 (3.9)	361 (3.3)	
Radiation first	9 (0.3)	16 (0.6)	10 (0.4)	17 (0.6)	52 (0.5)	
No treatment	115 (4.2)	62 (2.4)	62 (2.2)	82 (2.8)	321 (2.9)	

Data are expressed as *n* (%) unless otherwise specified

*ADI* Area Deprivation Index (ADI 1 = least deprived, ADI 4 = most deprived), *BMI* body mass index, *SD* standard deviation

There was a decrease in the proportion of privately insured patients from ADI 1 (78.5%) to ADI 4 (58.1%), and an increase in the proportion of Medicaid patients (ADI 1 = 0.9%, ADI 4 = 13.3%). The proportion of patients with Medicare, military, or no insurance was comparable across all ADI groups (Table 1).

### Association of ADI, Race and Ethnicity, and Insurance with Advanced Stage at Diagnosis

ADI was significantly associated with presenting with advanced stage at the time of diagnosis (*p* = 0.007). The odds of presenting with advanced stage increased with increasing neighborhood deprivation—women residing in ADI 4 had the highest odds of presenting with advanced stage (OR 1.48, 95% CI 1.19–1.84, *p* < 0.001) versus ADI 1 (reference) (Table 2).

Patients identifying as Black had the highest odds of presenting at advanced stage (OR 1.35, 95% CI 1.11–1.63, *p* < 0.001) versus those who identified as White (reference). Asian, Hispanic, and other patients were not found to be at increased risk for advanced stage at diagnosis, although the relative numbers for these groups were low (Table 2).

**Table 2:** Multivariate logistic regression analyzing the association of ADI, race and ethnicity, and insurance type with advanced stage (III/IV) at diagnosis among women with breast cancer at Penn Medicine, 2011–2019

Variables	Subcategories	OR	95% CI	*p*-Value	Composite *p*-value
ADI	1—least deprived	Reference			0.007
	2	1.27	1.02–1.57	0.030	
	3	1.31	1.06–1.61	0.011	
	4—most deprived	1.48	1.19–1.84	<0.001	
Race and ethnicity	White	Reference			0.022
	Asian	0.98	0.66–1.41	0.915	
	Black	1.35	1.11–1.63	0.002	
	Hispanic	1.01	0.66–1.48	0.978	
	Other	0.86	0.42–1.59	0.660	
Insurance type	Private	Reference			<0.001
	Medicaid	1.94	1.51–2.49	<0.001	
	Medicare	1.16	0.95–1.41	0.123	
	Military	0.27	0.02–1.28	0.200	
	Uninsured	2.21	1.27–3.67	0.003	
Age group, years	<40	Reference			<0.001
	40–70	0.47	0.37–0.60	<0.001	
	>70	0.41	0.31–0.54	<0.001	

*ADI* Area Deprivation Index, *CI* confidence interval, *OR* odds ratio

Insurance status was significantly associated with advanced stage at diagnosis (*p* < 0.001). Patients without insurance (OR 2.21, 95% CI 1.27–3.67, *p* = 0.003) and with Medicaid (OR 1.94, 95% CI 1.51–2.49, *p* < 0.001) were more likely to present with advanced stage than those with private insurance (reference) (Table 2).

Likewise, age was significantly associated with advanced stage at diagnosis (*p* < 0.001). Patients aged 40–70 years (OR 0.47, 95% CI 0.37–0.60, *p* < 0.001) and >70 years (OR 0.41, 95% CI 0.31–0.54, *p* < 0.001) were less likely to present with advanced stage than women <40 years of age (Table 2).

### Association of ADI, Race and Ethnicity, and Insurance with Treatment within 60 Days

Patients who identified as Asian (vs. White; OR 0.37, 95% CI 0.23–0.63, *p* < 0.001), had Medicaid insurance (OR 0.57, 95% CI 0.34–1.01) or no insurance (OR 0.25, 95% CI 0.13–0.57, *p* < 0.001) [both vs. private insurance], were >70 years of age (vs. <40 years of age; OR 0.35, 95% CI 0.15–0.70, *p* = 0.006), and presented with advanced stage (vs. early stage; OR 0.67, 95% CI 0.51–0.88, *p* = 0.003) were significantly less likely to receive treatment within 60 days (Table 3). However, compared with the least deprived patients (i.e., ADI 1, reference), those from more deprived neighborhoods were approximately twice as likely to receive treatment within 60 days (Table 3).

**Table 3:** Association of ADI, race and ethnicity, insurance type, and stage at diagnosis with adjusted odds of receiving treatment within 60 days at Penn Medicine, 2011–2019

Variables	Subcategories	OR	95% CI	*p*-Value	Composite *p*-value
ADI	1—least deprived	Reference			<0.001
	2	2.05	1.34–3.05	<0.001	
	3	2.10	1.44–3.10	0.001	
	4—most deprived	1.75	1.19–2.60	0.005	
Race and ethnicity	White	Reference			<0.001
	Asian	0.37	0.23–0.63	<0.001	
	Hispanic	1.11	0.48–3.23	0.822	
	Black	0.89	0.58–1.38	0.581	
	Other	0.19	0.13–0.38	<0.001	
Insurance type	Private	Reference			<0.001
	Medicaid	0.57	0.34–1.01	0.043	
	Medicare	0.91	0.64–1.29	0.567	
	Military	1.22	0.20–1.42	0.965	
	Uninsured	0.25	0.13–0.57	<0.001	
Stage at diagnosis	Early stage	Reference			0.004
	Late stage	0.67	0.51–0.88	0.003	
Age group, years	<40	Reference			<0.001
	40–70	0.67	0.30–1.31	0.286	
	>70	0.35	0.15–0.70	0.006	

*ADI* Area Deprivation Index, *OR* odds ratio, *CI* confidence interval

### Within-Group Analyses

Within ADI groups, race/ethnicity was found to be significantly associated with advanced stage at diagnosis for all ADIs except ADI 3. Within ADI 1, identifying as Hispanic was associated with advanced stage (OR 3.45, 95% CI 1.35–7.78, *p* = 0.005), while in ADI 2 and ADI 4, identifying as Black was significantly associated with advanced stage (ADI 2: OR 1.69, 95% CI 1.02–2.69, *p* = 0.033; ADI 4: OR 1.44, 95% CI 1.13–1.83, *p* = 0.003) (Table 4).

**Table 4:** Association between race and ethnicity and advanced stage at diagnosis within ADI groups at Penn Medicine, 2011–2019

Rank	Subcategories	OR	95% CI	*p*-Value	Composite *p*-value
ADI 1	White	Reference			0.049
	Black	1.40	0.25–3.05	0.359	
	Asian	1.00	0.53–1.76	0.977	
	Hispanic	3.45	1.35–7.78	0.005	
	Other	1.06	0.25–3.04	0.921	
ADI 2	White	Reference			0.042
	Black	1.69	1.02–2.69	0.033	
	Asian	1.37	0.62–2.68	0.392	
	Hispanic	0.00	0–488.19^a^	0.973	
	Other	1.74	0.50–4.65	0.320	
ADI 3	White	Reference			0.794
	Black	1.17	0.70–1.86	0.541	
	Asian	1.36	0.59–2.76	0.431	
	Hispanic	0.93	0.32–2.16	0.876	
	Other	0.44	0.03–2.16	0.430	
ADI 4	White	Reference			0.029
	Black	1.44	1.13–1.83	0.003	
	Asian	0.73	0.17–2.10	0.614	
	Hispanic	1.47	0.861–2.41	0.138	
	Other	0.65	0.10–2.25	0.566	

*ADI* Area Deprivation Index, *OR* odds ratio, *CI* confidence interval

^a^CI was large for this group due to the small sample size (*n* = 32)

## Discussion

In our retrospective analysis of patients at a large academic health system in the Philadelphia metropolitan area, we found that neighborhood deprivation, identifying as Black, and having Medicaid or no insurance were associated with advanced stage at time of diagnosis with breast cancer. Patients who identified as Asian, had Medicaid or no insurance, were >70 years of age, and presented with advanced stage were less likely to receive treatment within 60 days. Notably, while patients residing in neighborhoods with greater deprivation were more likely to present with advanced disease, they were actually more likely than patients from the least deprived areas to receive treatment within 60 days, which is increasingly felt to be the threshold for timely treatment initiation and is associated with improved survival.^17,18^ These findings suggest that while living in a deprived neighborhood may contribute to a patient presenting with advanced disease, once a diagnosis of breast cancer has been made, neighborhood deprivation does not necessarily predict delays in treatment.

In addition to the association between identifying as Black and presenting with advanced stage, our within-group analysis demonstrated that even among patients of deprived neighborhoods, Black patients were most likely to present with advanced stage; review of the literature reveals several reasons for this phenomenon. Black patients have been found to have more negative healthcare experiences, including discrimination from providers, which may dissuade patients from seeking care that would allow early detection.^19–21^ Similarly, several studies have demonstrated the relationship between the systemic racism routinely experienced by Black patients in healthcare settings and adverse health outcomes.^22–24^

Several studies have found associations between residing in deprived neighborhoods and pathological changes to the genetic sequence that may partially provide biological explanations for advanced stage at presentation among individuals in more deprived environments.^25–27^ As well as social factors, research suggests a predisposition to more aggressive disease phenotypes for patients of African ancestry. Specifically, the work by Newman and Kaljee revealed that a gene inherited from a specific West African ancestral group may help explain the greater burden of triple-negative breast cancer among Black patients in the US.^28,29^ Thus, although Black women participate in screening mammography at rates comparable with other groups, a combination of factors may still contribute to their increased likelihood of presenting with advanced disease.^30^ In addition, research recently published by our group suggests that despite comparable screening participation rates among Black and White women, Black women may be less likely to have timely work-up and diagnostic resolution of abnormal screening mammograms.^31^ Overall, the findings of our study and prior literature suggest that Black patients face additional factors, ranging from social to biological, that contribute to their increased likelihood, even among socioeconomic peers, of presenting with advanced disease.

Notably, within-group analysis in our study revealed that Hispanic patients were most likely to experience advanced stage among the least deprived patients (ADI 1). Prior studies have found Hispanic patients to have a higher burden of chronic disease and to be less likely to receive definitive treatment for many conditions.^32,33^ Nonetheless, Hispanic patients have also historically had both lower incidence and higher survival for many cancers, including breast, compared with White patients.^34^ Specifically for breast cancer, protective factors highly prevalent among Hispanic women were identified, including breastfeeding, younger age at first birth, and higher parity.^35,36^ However, the increased risk for late-stage presentation in ADI 1 patients suggests that protective factors usually attributed to this group may be more important among those with less socioeconomic advantage. In our study, the sample size for Hispanic patients was small compared with Black and White patients. In addition, the largest Hispanic communities in Philadelphia are relatively far from our institution, and its members often pursue care at other institutions within the city. Thus, it is possible that the Hispanic patients in our study are not especially representative of Hispanic women with breast cancer across the region. Likewise, our study is limited in the analysis of patients identifying as Asian, aggregating patients of diverse national backgrounds from more than 20 countries spanning East Asia, Southeast Asia, and South Asia under a single group.^37^ Asian women in our cohort were more likely to experience treatment delay than White women, a statistically significant finding despite the relatively small sample. The rise in the incidence of breast cancer in Asian women underscores the importance of more nuanced analyses and may suggest unmeasured disparities for Asian patients,^38^ which we have previously reported.^39–41^

### Limitations

A limitation of our study is that we were unable to assess the validity of ADI in predicting individual socioeconomic status, as the database used for this study did not collect individual patient income. In addition, our study had a relatively small sample of patients diagnosed at advanced stages compared with those diagnosed at an early stage, which may have confounded our analysis. Our sample sizes were also smaller for non-Black and non-White patients in this study, thereby limiting our ability to draw significant and nuanced conclusions for these populations.

Our study was a retrospective review, with expected selection biases, and was limited to one academic health system in a single, albeit large, metropolitan area. The patient population of our study, all treated at the University of Pennsylvania, may reflect a more advantaged population than the city of Philadelphia overall, as there was a greater proportion of patients living in ADI <50 neighborhoods compared with those living in ADI >50 neighborhoods. In addition, when assessing other measures of deprivation, our study had lower proportions of patients on Medicaid or uninsured patients when compared with the 2021 national average (Medicaid 5.3% vs. 18.9% nationally; uninsured 1.1% vs. 8.3% nationally) [42]. Although our population is diverse and the UPHS system includes multiple hospitals serving a wide catchment area, patient data from other peer institutions in the Philadelphia region might yield different findings that reflect the areas that those hospitals serve.

We excluded patients diagnosed outside of the UPHS to avoid selection bias. An exploratory analysis we conducted when designing our study demonstrated that patients presenting for second opinions following diagnosis at an outside hospital had a higher proportion of early-stage disease and lower ADI on average than the remainder of the cohort, suggesting a more privileged group whose inclusion might confound our findings. We realize doing so may of course also bias our study along other unforeseen dimensions.

## Conclusion

Our findings demonstrate that neighborhood deprivation was associated with advanced stage at diagnosis for breast cancer patients in Philadelphia. Interestingly, patients from deprived neighborhoods were more likely to receive treatment within 60 days than patients from least deprived areas. Within more deprived neighborhoods, Black patients were more likely to present with advanced stage at diagnosis, while in the least deprived neighborhoods, Hispanic patients were more likely. While Asian, older, uninsured, and late-stage patients were more likely to experience treatment delay, patients from more deprived neighborhoods were not. As neighborhood deprivation was associated with advanced stage at breast cancer diagnosis but not with greater likelihood of treatment delay, our findings suggest the opportunity for neighborhood-level opportunities in the greater Philadelphia area to improve screening and decrease rates of late-stage diagnosis.
